# Drought exposure and severe mental distress among adolescent girls and young women in Lesotho: the moderating role of food insecurity

**DOI:** 10.1186/s12888-026-08116-8

**Published:** 2026-04-28

**Authors:** Ololade Julius Baruwa, Oluwaseyi Dolapo Somefun, Hlengiwe Gwebu

**Affiliations:** 1 Department of Nursing and Public Health, University of Fort Hare, East London, Eastern Cape South Africa; 2 School of Public Health, University of the Western Cape, Cape Town, South Africa

**Keywords:** Drought exposure, Severe mental distress, Food insecurity, Adolescent girls and young women, Lesotho

## Abstract

**Background:**

Climate-related shocks such as drought are increasingly recognised as threats to mental health, yet evidence remains limited on how these stressors affect adolescent girls and young women (AGYW) in Lesotho. This study examines the association between drought exposure and severe mental distress among AGYW in Lesotho and assesses whether household food insecurity moderates this relationship.

**Methods:**

We analysed nationally representative cross-sectional data from 7,101 AGYW aged 13–24 years drawn from the 2018 Lesotho Violence Against Children and Youth Survey. Severe mental distress was measured using the Kessler-6 scale. Drought exposure was defined using district-level Integrated Food Security Phase Classification data. Multivariable logistic regression models assessed independent associations, and interaction terms were used to examine moderation by food insecurity. Predicted probabilities were estimated to illustrate combined effects.

**Results:**

Drought exposure was associated with increased odds of severe mental distress (adjusted odds ratio [aOR] = 1.47; 95% CI: 1.01–2.15; *p* = 0.045). Food insecurity independently increased the odds of distress (aOR = 1.47; 95% CI: 1.06–2.05; *p* = 0.022). In the interaction model, the association between drought vulnerability and severe mental distress differed by food insecurity status (interaction aOR = 2.21; 95% CI: 1.15–4.27; *p* = 0.018). AGYW experiencing both drought and food insecurity had the highest predicted probability of severe mental distress.

**Conclusions:**

The association between drought vulnerability and severe mental distress among AGYW in Lesotho was stronger in food-insecure households. Integrated climate adaptation, food security, and mental health interventions may help protect vulnerable AGYW in drought prone settings such as Lesotho.

**Supplementary Information:**

The online version contains supplementary material available at 10.1186/s12888-026-08116-8.

## Background

Mental distress is a primary contributor to global disability, with depression and anxiety being the most prevalent conditions, often with early onset among adolescents aged 14 [13]. Adolescent girls and young women (AGYW) are particularly affected, as this period represents a critical developmental stage characterised by rapid biological, psychological, and social transitions. Globally, mental health conditions disproportionately affect populations living in poverty, creating a bidirectional relationship between mental distress and socioeconomic disadvantage that is especially pronounced in low- and middle-income countries (LMICs), which bear an estimated 81% of the global mental health burden [8, 25]. In Africa, around 29 million people, or 9% of the population, experience depression [7]. The social and economic consequences of widespread mental illness are significant, including an estimated annual loss of US$1 trillion to the global economy due to reduced productivity from anxiety and depression [30].

The burden of mental distress is particularly consequential during adolescence and young adulthood, when disruptions can have lasting implications across the life course [1]. This developmental window encompasses key milestones, including brain maturation, educational transitions, the onset of puberty, entry into the workforce, and shifts in the social conditions that shape health [22, 28]. For AGYW, early onset of mental distress can disrupt educational attainment, limit economic opportunities, and may reduce long-term well-being, thereby reinforcing gendered inequalities across the life span [1, 12].

These risks are further exacerbated by environmental stressors, such as those induced by climate change [26]. Climate-related disasters, including droughts, floods, and extreme weather events, are recognised as potent risk factors for adverse mental health outcomes like anxiety, depression and mental distress [16, 34]. For AGYW, the mental health consequences of climate change are often indirect, operating through disruptions to household stability, schooling, caregiving and economic security. Food insecurity, in particular, represents a critical pathway through which environmental stressors may affect mental well-being as it can destabilise household dynamics, reduce parental or caregiver support and heighten uncertainty about the future [17]. These pathways are critically relevant in agrarian contexts, where livelihoods are closely tied to climate variability [21].

Lesotho, a small, mountainous, and agriculturally dependent country in Southern Africa, is highly vulnerable to increasingly severe droughts [6]. While the impacts on water availability and crop yields are well documented [3, 19], the psychological consequences, particularly among AGYW, remained less visible. Emerging research suggests that children, adolescents and young women may experience disproportionate mental health impacts from environmental stressors, as cumulative exposure to adversity during developmental periods can disrupt emotional regulation and coping capacities [35].

Critically, the mental health consequences of drought exposure are not uniform. Drawing on the stress-process model, the effects of environmental stressors are shaped by socio-economic vulnerability and access to buffering resources [31, 36]. In the context of Lesotho, drought may represent an existential threat for resource constrained households, directly undermining livelihoods and food security and intensifying mental distress among adolescents. However, those in more economically stable households may be partially buffered from these effects. Pre-existing inequalities, such as orphanhood or gender-related disadvantage, may further intersect with economic vulnerability to create layered risks [14]. Studies from sub-Saharan Africa have documented strong links between food insecurity and adolescent suicidality and psychological distress, particularly among those with limited social support [18].

Despite the growing recognition of the mental health implications of climate change, empirical research focusing specifically on examining the drought-mental health pathway among AGYW in Lesotho remains sparse. Existing studies in the region have largely focused on nutritional, educational or infectious disease outcomes, with limited attention to psychological well-being. This evidence gap limits the development of targeted and contextually appropriate interventions. This study, therefore, addresses two key research questions: First, what is the association between drought exposure and poor mental health among AGYW in Lesotho? Second, to what extent does food insecurity moderate this association? By addressing these questions, this study contributes to the existing literature on climate change, AGYW mental health, and social determinants of health. The findings are intended to inform targeted climate adaptation and mental health policies designed to protect the most socio-economically vulnerable youth, thereby advancing equity in a changing climate.

## Methods

### Study design and sample

This study used a secondary data analysis of the 2018 Lesotho Violence Against Children and Youth Survey (VACS) data. VACS is a cross-sectional household survey of males and females aged 13–24 years with the goal of producing nationally representative data on violence outcomes. Its main objectives were to define the scope and type of violence against children and youth, examine the health consequences, identify potential risk and protective factors, assess service utilisation, and inform prevention policies and programs. This study was a secondary analysis of existing survey data and did not involve any intervention or assignment of participants; therefore, it does not meet the definition of a clinical trial and was not subject to clinical trial registration. The 2018 Lesotho VACS was conducted in accordance with international ethical standards for research involving children and adolescents. The present study used de-identified secondary survey data, and no participants were directly recruited or contacted by the authors. Detailed information on the ethical approvals and consent procedures for the original survey is provided in the Declarations section.

The Lesotho VACS employed a multi-stage sampling design, which included the random selection of a cluster, probability systematic sampling of households within clusters, and random selection of a respondent (aged 13–24) from within the household. The VACS protocol adhered to WHO guidelines, requiring informed consent or assent from all participants. Minors participating in the survey required permission from their parents or guardians for the interviews, along with obtaining assent directly from the minors. This approach aligned with the established standards for research involving children. The VACS questionnaire covered topics on demographics, parental relationships, sexual history, and risk-taking behaviours. More information about VACS sampling methods, surveys, processes, and research ethics has been published elsewhere [20]. The study population for this analysis consisted of 13–24-year-old adolescents (*n* = 7101).

### Study measures

The variables used in this secondary analysis were selected from the original VACS questionnaire to align directly with the study objectives. Specifically, the analysis used severe mental distress as the outcome, district-level drought vulnerability as the main predictor, household food insecurity as the moderator, and sociodemographic and violence-related variables as covariates.

***Severe mental distress*** was assessed using the six-item Kessler Psychological Distress Scale (K6), which captures symptoms such as nervousness, hopelessness, restlessness, jumpiness, sadness, and feelings of worthlessness experienced in the past 30 days. Each item is scored on a five-point Likert scale, ranging from *0 (none of the time)* to *4 (all of the time)*. Total scores range from 0 to 24. Consistent with established cut-offs, a score of 13 or higher was classified as severe mental distress, indicating a level of symptomatology that may require clinical attention [24]. For the present analysis, we generated a binary outcome variable reflecting the presence or absence of severe mental distress based on this threshold.

***Drought exposure*** was operationalised using district-level classifications from the Integrated Food Security Phase Classification (IPC), an internationally recognised framework that assesses and classifies the severity of acute food insecurity associated with environmental shocks such as drought, crop failure, and climatic variability. To ensure temporal alignment with the survey period, we used the 2018 IPC Acute Food Insecurity Analysis for Lesotho, which assessed food security conditions during the same period in which the VACS survey was conducted. The IPC analysis identified several districts experiencing elevated levels of food insecurity associated with drought-related environmental stress. Based on these classifications, four districts, Maseru, Mohale’s Hoek, Qacha’s Nek, and Quthing, were identified as districts with heightened vulnerability to drought-related food insecurity. A binary variable was therefore created to capture drought exposure among AGYW, coding respondents residing in these districts as exposed (“yes”) and those residing in other districts as unexposed (“no”) [9].

***Food insecurity*** was measured using a household-level indicator reported by AGYW in the Lesotho VACS dataset. The measure is based on the survey question: “Do you think your household has enough money for food?” Responses indicating that the household did not have sufficient resources for food were used to classify AGYW as food insecure. For this study, AGYW from households reporting inadequate money for food were coded as “yes” (food insecure), while all others were coded as “no.”

### Covariates

We adjusted for eight covariates selected based on theoretical relevance and prior evidence on determinants of mental distress among AGYW [2, 5, 8]: age, marital status, school and employment status, orphanhood, household poverty, sexual violence, physical violence, and emotional violence. *Age* was categorised into two groups (13–17 vs. 18–24 years). *Marital status* was coded as a binary variable indicating whether the respondent had ever been married or had lived with a partner as though married at the time of the survey. *School and employment status* (*Not in school and not employed*) captured adolescents who were neither enrolled in school nor engaged in any form of work during the previous 12 months. *Orphanhood* was defined as the loss of one or both biological parents. *Household poverty* was measured using a composite wealth index provided in the Lesotho VACS dataset. The index incorporates indicators of household living conditions (type of drinking water, toilet and cooking facilities, construction materials for floors, roofs, and walls), asset ownership (e.g., television, car, motorcycle, land, bank account), ownership of livestock or farmland, and crowding (number of persons per room). A Principal Component Analysis (PCA) was used to generate wealth quintiles, ranging from quintile 1 (poorest) to quintile 5 (richest). For this study, respondents in the lowest two quintiles were classified as living in poverty. *Sexual violence* was assessed as any experience of unwanted, coerced, or physically forced sexual intercourse, or attempts at forced sex, perpetrated by partners, peers, family members, or community members in the past 12 months. Responses were coded as “yes” if any sexual violence was reported and “no” otherwise. *Physical violence* referred to experiences of being slapped, pushed, shoved, shaken, having hair pulled or arms twisted, being pinched, or having objects thrown at them by a partner, peer, family member, or community member in the past 12 months. This variable was coded dichotomously (“yes” vs. “no”). Emotional violence captured experiences of feeling unloved, unwanted, or undeserving of love from parents, caregivers, or relatives. This variable was similarly coded as “yes” if emotional violence was reported and “no” if none was reported.

### Data analysis

Initial data checks were first conducted to assess data completeness, internal consistency, and the distribution of all analytic variables. Missing data were evaluated across all variables, with the proportion of missingness ranging from 0% to 3.7%, with no systematic clustering of missingness across key predictors or outcome variables. Given the very low level of missing data, the potential bias introduced by complete-case analysis was considered minimal. In line with methodological guidance suggesting that listwise deletion is acceptable when missingness is small and unlikely to bias estimates, we retained this approach for multivariable analyses, ensuring that all estimates were based on fully observed data. All analyses accounted for the complex survey design of the Lesotho VACS using Stata 16 survey (svy) procedures, incorporating the sampling weight, primary sampling unit, and stratum variables supplied in the dataset to obtain nationally representative estimates and correct standard errors. Prior to fitting the multivariable models, we assessed multicollinearity among the predictor variables using the variance inflation factor (VIF) and tolerance statistics (Supplementary Table S1). No evidence of problematic multicollinearity was observed, with VIF values ranging from 1.01 to 1.50 (mean VIF = 1.18) and tolerance values ranging from 0.67 to 0.99, indicating acceptable independence among the covariates.

Data analysis proceeded in five stages. First, we generated weighted descriptive statistics for all key variables, including severe mental distress, drought exposure, food insecurity, and sociodemographic and violence-related covariates (age, marital status, school and employment status, orphanhood, household poverty, sexual violence, physical violence, and emotional violence). All estimates were weighted to account for the three-stage complex sampling design of the Lesotho VACS. Second, we examined bivariate associations between severe mental distress and each independent variable using chi-square tests, applying a significance threshold of *p* < 0.05.

Third, we fitted multivariable logistic regression models to assess the independent association between drought exposure and severe mental distress among AGYW, adjusting for all covariates in model (1) Fourth, to assess whether food insecurity modified the relationship between drought and mental distress, we conducted a moderation analysis by introducing an interaction term between drought exposure and food insecurity in the final adjusted model (model 2). To examine moderation, both drought vulnerability and food insecurity were treated as binary variables, and a multiplicative interaction term between them was included in Model (2) The statistical significance of the interaction term was formally assessed. Fifth, we estimated adjusted predicted probabilities to illustrate how the likelihood of severe mental distress varied across combinations of drought exposure and food insecurity, holding covariates constant. We analysed data from 7,101 AGYW aged 13–24 years in Lesotho for descriptive analyses. The multivariable models used a slightly smaller complete-case sample after excluding observations with missing data on model variables (Table 1). The final analytical sample consisted of 6845 AGYW.

## Results

### Characteristics of the study population

Overall, 4.0% of participants reported severe mental distress. Nearly 44.5% of AGYW resided in districts classified as drought-affected, while 65.9% experienced household food insecurity. More than half of the sample (57.3%) were young women aged 18–24 years, 23.7% were married or cohabiting, and 30.4% were not enrolled in school nor working at the time of the survey. Almost half (47.6%) had experienced orphanhood, and 40.0% lived in poor households. Among AGYW in our study population, 8.8% reported experiencing sexual violence in the past 12 months, 16.4% experienced physical violence in the past 12 months, and more than one-third (35.9%) reported emotional violence. Missing data were minimal across most variables, with the exception of orphanhood (3.7%), which remains within acceptable limits for population survey analyses.

**Table 1:** Study characteristics

Variables	Sample size: *n*/*N*	Percentage (95%CI)	Missing values
**Outcome**			
Severe mental symptoms	273/7101	4.0 (3.39–4.82)	0 (0.00)
**Key predictors**			
Drought exposure	2407/7101	44.5 (42.27–46.73)	0 (0.00)
Food insecurity	4613/7101	65.9 (63.73–67.95)	91 (1.28)
**Covariates**			
18–24 vs. 13–17	3690/7101	57.3 (55.80-58.85)	0 (0.00)
Married or cohabiting	1605/7101	23.7 (21.80-25.78)	6 (0.08)
Not in school and not employed	2088/7091	30.4 (28.28–32.58)	10 (0.14)
Orphan	3201/6836	47.6 (45.99–49.10)	265 (3.73)
Household poverty	2899/7101	40.0 (35.15–45.06)	0 (0.00)
Sexual violence	557/7096	8.8 (7.59–10.12)	5 (0.07)
Physical violence	1169/7101	16.4 (14.87–18.12)	0 (0.00)
Emotional violence	2460/7101	35.9 (33.56–38.30)	0 (0.00)

### Prevalence and bivariate analysis of factors associated with severe mental distress among AGYW in Lesotho

Table 2 presents the prevalence of severe mental distress across key characteristics of AGYW, along with results from chi-square tests assessing their bivariate associations. The prevalence of severe mental distress was higher among AGYW who reported drought exposure (56.4%) compared to those in non-drought districts (44.0%). Similarly, food insecurity showed a significant association: 72.6% of AGYW experiencing severe mental distress reported food insecurity, compared to 65.6% among those without mental distress. Older AGYW (18–24 years) showed a higher prevalence of severe mental distress (70.8%) than those aged 13–17 years (56.8%). About 57.4% of AGYW who experienced severe mental distress reported the loss of one or both parents, compared to 47.2% among their peers who never lost any parent. Contrary to expectations, household poverty was less common among those with severe mental distress (32.5%) than among those without (40.3%), a pattern that may reflect mediation through more proximal stressors such as food insecurity and exposure to violence.

Experiences of violence demonstrated some of the strongest associations. Sexual violence was reported by 28.4% of AGYW with severe mental distress, compared to only 7.9% of those without distress. Similarly, physical violence (35.1% vs. 15.6%) and emotional violence (67.1% vs. 34.6%) were more prevalent among those experiencing severe mental distress. All three forms of violence showed highly significant associations. Marital status and school enrollment showed weaker associations. While married or cohabiting AGYW appeared slightly more likely to experience severe distress (26.8% vs. 23.6%), the association was not statistically significant. School non-enrollment approached significance, with higher distress among those not in school nor employed (37.1% vs. 30.1%).

**Table 2:** Bivariate Chi-Square analysis and prevalence of severe mental distress in Lesotho

Severe mental distress			
Variables	No	Yes	χ^2^ (*P*-value)
	Percentage (95%CI)	Percentage (95%CI)	
Drought exposure	44.0 (41.77–46.24)	56.4 (47.55–64.79)	**17.097 (0.006)**
Food insecurity	65.6 (63.46–67.65)	72.6 (65.64–78.69)	**6.024 (0.037)**
18–24 vs. 13–17	56.8 (55.17–58.34)	70.8 (63.76–76.98)	**22.233 (< 0.001)**
Married or cohabiting	23.6 (21.66–25.66)	26.8 (20.53–34.24)	1.587 (0.331)
Not in school and not employed	30.1 (27.99–32.32)	37.1 (30.01–44.81)	6.394 (0.053)
Orphan	47.2 (45.55–48.78)	57.4 (50.98–63.58)	**11.294 (0.002)**
Household poverty	40.3 (35.44–45.40)	32.5 (23.91–42.39)	**7.078 (< 0.001)**
Sexual violence	7.9 (6.78–9.29)	28.4 (21.78–36.15)	**144.456 (< 0.001)**
Physical violence	15.6 (14.15–17.26)	35.1 (27.18–44.01)	**76.299 (< 0.001)**
Emotional violence	34.6 (32.26–36.98)	67.1 (59.21–74.15)	**126.853 (< 0.001)**

### Associations between drought, food insecurity and severe mental distress

In the multivariable analysis (Table 3), Model 1 examined the independent associations of drought exposure and food insecurity on severe mental distress while adjusting for all covariates, and both drought and food insecurity emerged as significant predictors. AGYW living in drought-affected districts had 47% higher odds of reporting severe mental distress compared to those in non-affected districts (aOR = 1.47, 95% CI: 1.01–2.15; *p* = 0.045). Similarly, AGYW from food-insecure households had 47% increased odds of severe mental distress relative to those who were food secure (aOR = 1.47, 95% CI: 1.06–2.05; *p* = 0.022).

Among AGYW, those who experienced sexual violence in the past 12 months were more likely to report severe mental distress compared to those who did not experience sexual violence in the past 12 months (aOR = 2.52, 95% CI: 1.66–3.83; *p* < 0.001). Similarly, those who experienced physical violence in the past 12 months were more likely to report severe mental distress compared to those who did not experience physical violence in the past 12 months (aOR = 1.85, 95% CI: 1.26–2.72; *p* = 0.002). AGYW who reported ever experiencing emotional violence were more likely to report severe mental distress compared to those who never experienced emotional violence (aOR = 2.50, 95% CI: 1.76–3.56; *p* < 0.001). All other covariates remained insignificant.

### Moderating effect of food insecurity on the drought–mental distress relationship

Model 2 examined whether household food insecurity modified the association between drought exposure and severe mental distress by including an interaction term between drought exposure and food insecurity. The interaction term was statistically significant (aOR = 2.21, 95% CI: 1.15–4.27, *p* = 0.018), indicating that the association between drought exposure and severe mental distress differed according to household food security status.

In the same model, the main effects represent associations at the reference level of the interacting variable. Accordingly, the coefficient for drought exposure reflects the association among AGYW living in food-secure households, while the coefficient for food insecurity reflects the association among those not exposed to drought. After inclusion of the interaction term, the main effects of drought exposure *(aOR = 0.83*,* 95% CI: 0.45–1.52*) and food insecurity *(aOR = 0.95*,* 95% CI: 0.64–1.42)* were not statistically significant at these reference levels.

**Table 3:** Multivariable logistic regression analysis of severe mental distress among AGYW in Lesotho

	Model 1 (adjusted OR)		Model 2 (adjusted OR)	
	aOR (95% CI)	*P*-Value	aOR (95% CI)	*P*-Value
Drought exposure	**1.47 (1.01–2.15)**	**0.045**	0.83 (0.45–1.52)	0.545
Food insecurity	**1.47 (1.06–2.05)**	**0.022**	0.95 (0.64–1.42)	0.809
Drought##food insecurity			**2.21 (1.15–4.27)**	**0.018**
18–24 vs. 13–17	1.50 (0.96–2.33)	0.074	1.51 (0.97–2.34)	0.068
Married or cohabiting	0.83 (0.55–1.25)	0.360	0.83 (0.55–1.25)	0.360
Not in school and not employed	1.38 (0.96–1.98)	0.082	1.40 (0.98–2.01)	0.068
Orphan	1.30 (0.98–1.72)	0.072	1.30 (0.98–1.73)	0.067
Household poverty	0.76 (0.51–1.12)	0.163	0.76 (0.52–1.13)	0.171
Sexual violence	**2.52 (1.66–3.83)**	**< 0.001**	**2.54 (1.66–3.87)**	**< 0.001**
Physical violence	**1.85 (1.26–2.72)**	**0.002**	**1.88 (1.28–2.76)**	**0.001**
Emotional violence	**2.50 (1.76–3.56)**	**< 0.001**	**2.49 (1.75–3.54)**	**< 0.001**

### Predicted probabilities of severe mental distress by drought and food insecurity

**Fig. 1:**
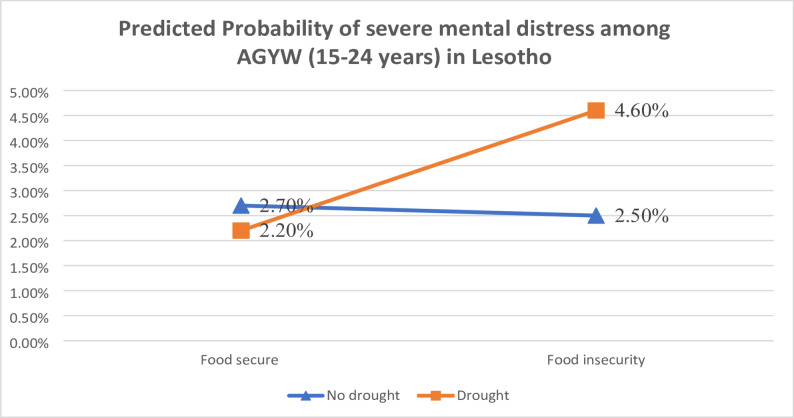
Predicted probability of mental distress by drought exposure and food insecurity among AGYW in Lesotho

Figure 1 presents adjusted predicted probabilities of severe mental distress across combinations of district-level drought vulnerability and food insecurity. The lowest predicted probability was observed among AGYW living in drought-vulnerable districts who were food secure (2.20%), whereas the highest predicted probability was observed among AGYW living in drought-vulnerable districts who were also food insecure (4.60%). These predicted probabilities are presented to complement the odds ratios and illustrate the joint association of drought vulnerability and food insecurity with severe mental distress in absolute terms.

## Discussion

This study provides empirical evidence that drought exposure and household food insecurity are significantly associated with severe mental distress among adolescent girls and young women (AGYW) in Lesotho. Importantly, our findings highlight that food insecurity moderates the relationship between drought and mental distress, with the co-occurrence of both conditions associated with increased risk of poor mental health outcomes. These results contribute to the growing body of literature on climate-related mental health impacts, particularly in low- and middle-income countries like Lesotho.

Our first key finding, that drought exposure is independently associated with a 47% increase in the odds of severe mental distress, aligns with the stress process model and prior research emphasising how environmental stressors can disrupt psychosocial well-being, especially in agrarian contexts [5, 29]. It is important to interpret this and all subsequent findings in light of the relatively low prevalence (4.0%) of severe mental distress in the study population. This low rate suggests that, while the relative odds increases associated with drought and food insecurity are substantial, the absolute risk for any individual remains modest within this nationally representative sample. Nonetheless, severe mental distress is an event that warrants immediate clinical attention [24]. As such, these associations remain clinically and public health relevant, as they identify specific, policy actionable risk factors that increase the likelihood of a high burden condition, even if that condition is not endemic.

Second, we found that food insecurity also independently increased the odds of severe mental distress by 47%, which is in line with other studies globally [4, 15, 32] and in African settings [10, 11]. This underscores a potential material pathway through which environmental shocks affect mental health: economic strain and perceived hunger may contribute to psychological distress. Notably, our moderation analysis revealed that food insecurity was associated with a stronger drought mental distress association in the interaction model. When both conditions are present, the predicted probability of severe mental distress rises to 4.60%, compared to 2.20% for those exposed to drought but remaining food secure. This finding highlights a vulnerable subgroup where interventions could yield a meaningful impact. The interaction suggests that drought’s psychological toll is not merely a function of environmental exposure but is critically mediated by socioeconomic vulnerability, specifically, the household’s ability to maintain food security.

These findings support the theoretical framework presented in the introduction, which posits that socio-economic vulnerability moderates the impact of environmental stressors on mental health [23, 27]. In Lesotho, where many households depend on rain-fed agriculture, drought can quickly translate into food insecurity, especially for asset poor families. This dual burden may reflect layered vulnerability in which climate-related strain and household deprivation co-occur in ways that are associated with poorer mental health.

Our study also reinforces the strong association between violence, sexual, physical, and emotional and severe mental distress, which remained robust across models. The magnitude of these associations (aORs ranging from 1.85 to 2.54) was notably larger than for drought or food insecurity alone, emphasising that interpersonal trauma may be a more potent proximal risk factor for severe distress in this population, even against the backdrop of environmental and economic stress.

Interestingly, household poverty, as measured by a wealth index, was not independently associated with increased mental distress in adjusted models. In the bivariate analysis, it was less common among those with severe distress. This counterintuitive finding may reflect the complex nature of poverty measurement or indicate that other, more acute forms of deprivation, such as food insecurity or violence, are more direct drivers of psychological distress. Alternatively, it may suggest that the effects of economic poverty are fully mediated through these more proximal variables in our model.

## Limitations

Several limitations should be considered. First, the cross-sectional design precludes causal inference. Second, drought exposure was measured at the district level, which may not capture micro-level variability in drought impact or individual exposure. This approach was necessitated by data availability, as no individual-level drought measures exist in the VACS dataset. Future research should incorporate finer-grained drought measures, such as household-reported impacts or GPS-linked climate data, to reduce this bias. Third, our measure of food insecurity was based on a single, subjective question regarding perceived household sufficiency of money for food. While practical, this measure lacks the multidimensionality and validation of more comprehensive food security scales and may not fully capture the experience of hunger, dietary quality, or anxiety about food. Fourth, mental distress was assessed using a screening tool (K6), not a clinical diagnosis. Adolescent boys and young men (ABYM) were not included in the analysis due to their low representation in the VACS dataset and the very low prevalence of reported severe mental distress among male respondents. As a result, we were unable to conduct meaningful gender-stratified analyses or directly compare mental distress outcomes by sex. Fifth, because the VACS is a household-based survey, AGYW who were homeless, institutionalised, or otherwise outside sampled households were not represented, which may limit generalisability to the most marginalised populations. Finally, although we adjusted for key covariates, residual confounding from unmeasured factors remains possibleic focus on safeguarding food security in drought-affected regions.

## Implications

Despite these limitations, the findings have important policy implications. They highlight the need to integrate mental health support into climate adaptation and humanitarian responses, with a specific focus on safeguarding food security in drought-affected regions. Scaling up access to food aid, cash transfers, and climate-resilient agricultural programs could buffer households from drought-induced food insecurity, the key moderator identified in our study, and thereby reduce downstream mental health risks for AGYW. The identification of a high-risk subgroup AGYW experiencing both drought and food insecurity provides a clear target for layered interventions that combine food assistance, economic support, and accessible mental health services. Furthermore, the robust link with violence underscores the necessity of integrating violence prevention and response into youth mental health and climate resilience programs.

Future research should employ longitudinal designs to establish temporal order and explore protective factors. Qualitative studies could illuminate the lived experiences of youth navigating these adversities. Research using more nuanced measures of both food security and mental health outcomes would help clarify the pathways identified here.

## Conclusion

This study advances understanding of how climate-related stressors interact with socioeconomic vulnerability to shape mental health outcomes among youth in Lesotho. By demonstrating that food insecurity significantly moderates the association between drought exposure and severe mental distress, our findings underscore that the mental health consequences of climate shocks are not uniform but are amplified under conditions of material deprivation.

These findings have implications for climate-vulnerable settings beyond Lesotho, particularly across sub-Saharan Africa, where similar agrarian economies face recurrent drought. Our results are most likely to generalise to contexts that share Lesotho’s key characteristics: dependence on rain-fed agriculture, limited household adaptive capacity, high baseline food insecurity, and layered vulnerabilities among adolescent girls and young women. Neighbouring Southern African countries that declared drought emergencies in 2024, including Botswana, Zambia, and Namibia, exemplify settings where the moderating pathway we identified may be similarly operative [33]. However, generalizability is not automatic. Our findings may not extend to urban settings, economies with diversified livelihoods, or contexts with robust social protection systems that buffer drought-induced food insecurity. Future research should employ comparable methods in other African countries to establish whether the drought-food insecurity-mental health pathway we observed represents a general phenomenon or reflects Lesotho-specific dynamics. For now, our findings offer a theoretical framework grounded in the stress-process model that can guide hypothesis testing in other contexts while acknowledging that boundary conditions matter.

These findings suggest three priority areas for intervention. First, social protection programmes, particularly unconditional cash transfers are important, as they have been shown in sub-Saharan Africa to improve both food security and mental health outcomes. Expanding programmes like Lesotho’s Child Grants Programme to drought-affected districts could buffer the mental health impacts we observed. Second, school feeding programmes represent a directly targeted intervention for AGYW as shown elsewhere. Third, integrated mental health services should be embedded within climate adaptation responses, including community-based psychosocial support and youth-friendly services.

## Supplementary Information

Below is the link to the electronic supplementary material.

Supplementary Material 1

## Data Availability

The datasets analysed are available upon request at: [https://www.togetherforgirls.org/resources/lesotho-violence-against-children-and-youth-survey-vacs/] (https://www.togetherforgirls.org/resources/lesotho-violence-against-children-and-youth-survey-vacs).
