# Supplementary material for: Drought exposure and severe mental distress among adolescent girls and young women in Lesotho: the moderating role of food insecurity
Source: BMC Psychiatry. 2026 Apr 28;26:464. doi: 10.1186/s12888-026-08116-8 (PMC13267369; doi:10.1186/s12888-026-08116-8)
Supplement: Supplementary file 1 — Supplementary Material 1 [file 12888_2026_8116_MOESM1_ESM.docx]

**Supplementary**

**Table S1:**  Variance inflation factor and tolerance in the multivariable regression analysis

|  | **VIF** | **Tolerance** |
| --- | --- | --- |
| Variables |  |  |
| Drought exposure | 1.01 | 0.991785 |
| Food insecurity | 1.09 | 0.917890 |
| Age | 1.35 | 0.740854 |
| Ever married | 1.50 | 0.665924 |
| Not in school and not employed | 1.40 | 0.715497 |
| Orphan | 1.04 | 0.963709 |
| Household poverty | 1.14 | 0.878825 |
| Sexual violence | 1.07 | 0.931740 |
| Physical violence | 1.11 | 0.902848 |
| Emotional violence | 1.14 | 0.877608 |
| Mean VIF | 1.18 |  |
